# Treatment of Dental Pulp, Preparatory to Plugging

**Published:** 1851-04

**Authors:** J. D. White

**Affiliations:** Dentist.


### ARTICLE VIII.

*Treatment of Dental Pulp preparatory to Plugging.* By J. D. White, Dentist.

Arsenious acid *is*, undoubtedly, the *destroying agent* in every form in which it can be used. A great many dentists are now using it, combined with morphia, and with success. I cannot understand why the preparations of morphia will not obtund, to some extent, sensibility in the pulp of a tooth. They are applied, with that view, to other parts of the body,* and not without good effect. I know well that the sulphate of morphia alone will suspend pain in the pulp, when it only arises from inflammation of that substance. I employ it constantly for that purpose, and sometimes combined with tannic acid; and always succeed in stopping the pain, even though it has been occasioned by the application of arsenious acid alone, if the external membranes are not much involved.

*Arsenious Acid, Morphia and Kreosote.*—This is, perhaps, the best form of using arsenic that has yet been devised. I have not seen it spoken of by any authors. I have been using it thus since 1840, as a general substance for destroying the pulp, but I have tried arsenic in various other forms, as well as many other substances, before and since that period. There is no difference, in this form, in the rapidity with which it unites with the pulp, than when the kreosote only is properly combined with it; but the morphia will exert its narcotic influence, and lessen the pain; an effect which we do not often obtain with kreosote. The kreosote cannot be regarded in any other light than as a mere vehicle for the proper application of the arsenic.

℞ Arsenious acid, . . gr. xxx.
Morphia sulphas, . gr. xx.
Kreosote, q. s.
Misce.

* "They are applicable to all cases where the object is to relieve pain, or allay nervous irritation in any shape."—*U. S. Dispensatory.*

Put the arsenious acid and kreosote in a glazed mortar, and grind until the arsenic becomes impalpable; then add the sulph. of morphia, and continue the trituration for some time, with a view of completely incorporating both ingredients, and adding a little kreosote to keep the mass of about the consistency of thick cream. Prepared in this way, the arsenic is in a better condition to unite speedily with the pulp than the mere dry powder of arsenic, on account of the kreosote holding a large quantity of it in solution; and it becomes more minutely subdivided when triturated in an oily substance, than in the dry state.

*Manner of Applying the Paste.*—In the application of this paste, or in fact of any substance for the treatment of the pulp, great care should be taken to free the cavity of decay of all foreign substances, as well as the decay immediately over the pulp cavity, so as to be able to place the paste in immediate contact with the exposed pulp. This precaution should never be neglected; because, if the pulp is inflamed at the time the application is made, the simple removal of the decay will excite bleeding, and relieve, or wholly stop, the pain; indeed, it is very frequently that nothing more is required to cure a bad attack of tooth-ache. And again: if it be not inflamed at the time, the action of the destroying agent will excite the determination of blood to the pulp, and by being thus congested in a shut cavity, and incapable of expanding or bleeding, will produce great pain, wholly independent of the escharotic agent; whilst, on the other hand, the patient would only experience a gnawing sensation, or dumb pain. A pledget of cotton, about the size of a small pin's head, steeped in the paste, is sufficient. If the pulp bleed when the cavity is cleansed, we must wait until the bleeding subsides before we apply it, as it would dilute the preparation and diminish its action. The cavity may then be filled with cotton, and left to remain in from ten to sixteen hours. If it be in the case of a young patient, the bone will absorb a sufficient quantity of the arsenic to inflame the alveolo-dental membrane; and of course it should be removed, in such cases, in a shorter time than it could be left with safety in the case of an older patient, or a dense and opaque tooth.

I sometimes place a layer of tin foil over the paste after it is introduced, with a view of preventing it from being absorbed by the cotton, especially if it be between two teeth, both of which are decayed, and it is not desirable to destroy the pulp in both. We have seen cases where substances have been placed in one tooth to destroy the pulp, while the adjacent tooth was not decayed to the nerve; and the cotton absorbing the poison, it would pass over to the adjacent tooth, and permeate the thin stratum of bone protecting the pulp, and either inflame or destroy it, and give both patient and operator great trouble.

Softened beeswax and pastes of various kinds are objectionable, because they will not allow the air to escape from the cavity while packing them in, and therefore, by forcing a column of air against the pulp, it induces pain. Where the cavity cannot be sufficiently well shaped to allow of the secure package of the cotton, or where there is no adjacent tooth to support it, as on the labial surfaces of the teeth, for instance, I am in the habit of placing a roll of cotton over the cavity, and then throwing a ligature around the tooth to secure it.

An escharotic ought never to be applied in the after part of the day, or at night, to destroy the dental pulp, and especially in patients of a high nervo-sanguine temperament; because teeth are more liable to pain at night, from the increase of the nervous susceptibilities, and the febrile exacerbation and determination of blood to the head and face, that all are more or less liable to as night approaches. I very frequently narcotise the pulp, by applying morphia for a day or two before the application of the paste, if we fear its giving pain, and apply the escharotic in the morning. By this method, the most happy results have been produced in the treatment of the most nervous patients. Some of the reasons why I prefer using the arsenious paste are, 1st. It destroys the vitality of the pulp in a shorter time, and with less severe pain, than in any other form in which I have used it. 2d. It less frequently causes inflammation of the external membranes than when applied alone, from the well known principle, that the more speedily it unites with and produces the death of a part, the less extensively will it be absorbed: and, 3d. It produces a more extensive and perfect slough of the pulp, and of course favors its removal more effectually from the roots without pain. It is upon this latter effect that the preservation of the tooth mainly depends; and it would seem that dentists have pretty generally overlooked this necessity, under a false idea that the tooth is thereby rendered inevitably a foreign body, and consequently becomes itself a cause of exciting inflammation. I consider the actual cautery as preferable for removing the pulp, where it can be applied, to the use of acids, and often use it in treating the pulp, preparatory to setting pivot teeth.

*Manner of Removing the Pulp.*—After the paste has been in the allotted time, see the patient, and remove it. Then, with a small pointed instrument, wound the pulp to excite bleeding, to relieve the tension of the blood-vessels of the apex of the fang, and prevent the pain that would otherwise be produced by enlarging the orifice leading from the cavity of decay to the pulp cavity. Now open the orifice of the internal cavity well, quite as large as the largest part of the pulp cavity; then, with an annealed wire fitted into an ordinary drill-stock for the purpose, and bent to suit, and *barbed* with a sharp knife, so that when it is passed into the canal alongside of the pulp, this sharp, jagged instrument will lay hold of the mass of the pulp, and with a sudden jerk, very probably, the whole pulp will be extracted, or at least it will break off at the line of demarcation between the living and dead parts. If this barbed instrument be as pointed as possible, it will pierce the pulp, or pass along the canal to any desirable extent, without pushing the pulp, or the contents of the canal of the root, before it; and again, if the sharp teeth lay backwards, or towards the shaft of the instrument, they will not produce much obstruction in entering, but on withdrawing the instrument, they will inevitably bring with it the contents of the canal or cavity. If this instrument be filed to a flat square, and the edges barbed, they will be sharper than if the teeth were cut on a round or flat surface. To pass deep into the root through a winding cavity, where a steel instrument cannot well be used, a common quill, of hard texture, prepared like the steel instrument, will be useful, as it is more flexible. We use the quill entirely for mopping out the root, in washing away the blood, and putting any substance deep into the root. I do not often apply the paste a second time, especially if the pulp is destroyed to a considerable extent by the first application, but apply a small quantity of *caustic potash* or *chloride of zinc*, as either of those substances will produce a speedy slough, in this condition of the pulp, without pain, whilst if either be applied to the pulp in a healthy state, they will excite intense pain, and not destroy the pulp for any depth. I also use *burnt alum*, *tannic acid*, or *nitrate of silver*, in solution, or in the stick, as the circumstances of the case and the locality of the tooth would allow; as, for instance, it would be improper to use nitrate of silver in a front tooth, as it would, without great care, discolor it. Sometimes, if all the pulp cannot be taken away, by waiting a few days, the balance will slough, and it can be removed with facility. It is yet a question with me, whether the pulp should be removed to the very apex of the root, or only within a short distance of it. We know well that the only evil of removing the pulp is the wounding or communicating inflammation to the external membranes, and it seems to me that if we remove the pulp to the end of the root, and there is any irritability in the constitution against us, it must act upon the external membranes at once; and that approaching so close to the apex, we cannot avoid exciting some irritation, whilst, if we leave a small portion of the blood-vessels remain, say an eighth or quarter of an inch, as the canal in the root is wide or narrow, as a kind of neutral ground to work on, in this way we would not be coming close enough to the external membranes to produce irritation, and there would not be sufficient of the fragments remaining to do harm. Neither do I see why we could not get rid of so small an amount by absorption, as the external membranes could do without it, in the same way that we get rid of the small shreds of blood-vessels elsewhere under similar circumstances. Although, in removing several nerves in the same mouth, I have had trouble with those that have been partially removed, and not with those that had been entirely, and vice versa, still I give the preference to leaving some neutral ground. Yet I do not leave sufficient to invite an afflux of blood to the parts, or any part that can be taken away with the smallest flexible probe.

*Plugging the Canal of the Root or Nerve Cavity.*—I do not think it prudent to plug the root as soon as the nerve is removed, on account of the bleeding that generally follows, even though there be no bleeding; because it is evident that the blood that returned through the pulp must now return by anastomosing vessels, and which will give more or less turgescence to the blood-vessels of the root, and become augmented by the pressure of plugging. It is therefore important to wait for several days, as the case may be; in the meantime, see the patient, in order to remove the clot of blood that forms in the cavity until the bleeding ceases; and then, at last, fill the whole root with a tent of cotton imbued with alum-water, for a day or two, upon the withdrawal of which, if there be no bleeding, the root may be filled with gold. I *never* use tin in the root of a tooth: some I fill in three days, and some in two weeks, depending upon the condition of the external membranes. I take a piece of gold leaf, cut it triangular, (No. 6 will do, but 15 is better, and beginning at one corner, roll it into a pointed roll, and as hard as possible; this will make a flexible gold wire, which can be passed, if necessary, to the apex of the root. If it be too pointed or sharp, cut it off with the scissors at such point as to be thick enough to choke the cavity before it gets to the fragments of remaining blood-vessels, or going through the foramen at the apex of the root, for it would, in either case, bring on inflammation. Then follow this with a small annealed plugger, having a great number to fit different localities, of higher or lower temper, as may be desired, the larger ones to go far into the root, to be fitted extemporaneously, and used until a harder and stronger one will apply, making still greater and greater pressure as we near the neck of the tooth, when a very strong instrument must be used, as the plug at this point must be very hard, to prevent the tooth from becoming discolored, it ought to be hard to the end of the root; but I know of no way of getting an instrument in, strong enough to make it very hard in some cases. Now the first of these pluggers must be rather soft; still, not as soft as steel can be, and rubbed with a burnisher towards their points, as that process will lay a kind of burr towards the points, and when used will carry the gold in, and not withdraw it on removing the instrument. It will also harden those sufficiently that are to be used first, without plunging them into water. When the root of the tooth is filled level with the floor of the cavity of decay, I am in the habit of burnishing the surface of the plug, to shut off the possibility of dampness escaping into the external plug. It is obvious that if the natural cavity of the tooth be firmly plugged with gold, the tooth will be in a better state of preservation than if the cavity be open or plugged with any other substance, as it will effectually prevent the dampness, pus, air, &c., from acting on the walls of the cavity. I need not say what nastiness gets into that cavity, as once cleaning it out when the tooth is in a diseased condition will explain it much better than I can. Again, as the tubuli of the body of the tooth radiate from the pulp cavity to its periphery, an impervious plug will shut off the discoloration so much complained of when the pulp is destroyed, as all that so much dreaded "blueness," "purple," &c., is from infiltration of the tooth by the contents of the pulp cavity. In this way, the whole body and root is saturated by the morbid fluids bathing that cavity, until the whole tooth is dead. I never think of a tooth becoming discolored, if my patient will give it as much attention as I propose to give. It therefore never happens, except by carelessness on my part, my patients', or imperfect operations.

I copy the following from my Thesis paper, written in the winter of 1843–44. It may not be uninteresting to give a list of cases which I kept during April and May of 1842. In one hundred successive cases, the pulps were destroyed in eighty-four without pain; the remaining number, sixteen, gave pain, the average duration of which was one hour. The pain was most severe, and of greatest duration, in patients of a strong nervo-sanguine temperament; but even in those cases, if the pulp had been subject to frequent attacks of inflammation, it rarely gave pain when the paste was applied. Again, patients of scrofulous diathesis rarely suffered pain, whether the pulp had been previously subject to inflammation or not. I have extracted six of the above one hundred cases since the spring of 1842, for alveolar abscess, (time about twenty-two months,) but I was not able to trace the whole number any further, to obtain the ratio. Some, however, are still good, (1850,) and those that have been lost had not been well plugged in the roots. I never could succeed in saving teeth satisfactorily by plugging over the nerves, either by "caps," made according to special patterns, or methods of plugging, or interposition of non-conductors or non-irritating substances, such as *asbestos*, *charcoal*, *cotton*, &c. &c. I have kept a diary of cases, which will be useful to refer to, as well for my own practice as for others. The last special list was kept during October and November, 1849, of seventy cases that had been plugged over the nerves, and numbers of them by some of the most careful operators in Philadelphia, as well as in neighboring cities; none of them have been extracted up to this time, after filling in the roots, to my knowledge. Nearly all of those cases were giving pain at the time, and some of them had gone on to alveolar abscess, and were in a very unhealthy state. But this would lead to the consideration of alveolar abscess, which was not contemplated in the limits of these papers, but which, if time can be spared, and health permit, I will take up at some future period. I cannot close without acknowledging myself under the deepest sense of gratitude to the proprietors of the "Dental News Letter," and those other valuable journals that have done me the honor of publishing the above hastily written papers. Though hastily written, they are not hasty conclusions, as I have given them the most deliberate reflection and experiment, and their strict observance lead me daily to the most happy results. My humble exertions have been, and ever shall be, to arrive at the best and most truthful methods of alleviating human suffering, and I would earnestly and respectfully solicit every fellow-laborer in our useful avocation, to aid in the elucidation of this intricate subject. He who corrects most of my errors, and teaches me most, does me more service and honor than he who adopts and applauds the result of my labors.—*Dent. News Letter.*

